# Supplementary material for: Examining the foreign policy attitudes in Moldova
Source: PLoS One. 2021 Jan 15;16(1):e0245322. doi: 10.1371/journal.pone.0245322 (PMC7810516; doi:10.1371/journal.pone.0245322)
Supplement: Attachment — Submitted filename: Response to reviewers November 2020.docx [file pone.0245322.s002.docx]

**Response to reviewers**

**Reviewer #1: General Comments**

1.1 When the two-level variance components model is explained in section 3. Methods, the authors provide a description of all components of equation (1), except the component referred to the group level residual. Although this becomes perfectly clear when you continue reading the work, it is convenient to specify it the first time it is cited.

*R. I introduced a short explanation about the group level error (eq.1, section 3)*

1.2 In the paragraph in which the main variables of interest are described (section 4. Ethno-geopolitical context and Data, in the section dedicated to data), it would be interesting to directly justify the variables chosen according to the academic literature referred to in the previous sections.
I want to point out a minor format matter in the last paragraph of the third section (Methods) since there is a different font format to the rest of the paper.

*R. I introduced a new paragraph (p.10) to justify the selection of variables according to the literature. The selection of the four dependent variables as vectors of foreign policy is extensively motivated in several sections as Introduction and the first sub-section of section 4 (entitled The substance of foreign policy attitudes in Moldova).*

*I checked the last paragraph of the third section (Methods) and I didn’t find a different font format to the rest of the paper (TNR12).*

**Reviewer #2:**

- The title of the paper is misleading based on the scope of the article.

*R. I reformulated the title of the paper.*

- Abstract is hard to read. It does not provide a clear path of what the paper wants to achieve.

*R. I reformulated some parts of the Abstract.*

- Please, provide more details regarding data availability within the manuscripts and supporting information files. This is not clear at all.

*R. A large paragraph about the data availability and dataset description is the first one at the beginning of section Data (p.9). The dataset is freely provided online by the Institute for Public Policies in Moldova (as also mentioned in the paper).*

- Kindly, provide a brief context on the Transnistria conflict within your article. Why the conflict was not briefly described in the paper?

*R. I included a brief presentation of the Transnistria conflict at p.9 of the section “The substance of foreign policy attitudes in Moldova”.*

- Page 8: Attention to details. References used in the preparation of the paper were not found in the references section. For instance, See Baltag (2013)

*R. Yes, I corrected.*

- Page 8: It is not clear to readers, what is the pro-poor economic growth of 5%. Please, clarify or add more details.

*R. A footnote was added to provide an additional explanation in this regard.*

- Page 8: What do you mean by regional perspective? At this stage, It is not very clear to readers. Please, provide more details.

*R. An additional explanation was added in Introduction at p.3.*

- Typo on page 8: "the most important ethnic groups in Moldova ae Romanians"

*R. I made the correction.*

- Page 10: "but rather as latent values explaining the factor loadings according to the factor analysis". Kindly, provide more details, since it is not clear to readers.

*R. I reformulated a little bit for more clarity.*

- Page 12: " then the use of multilevel models becomes totally justified" Add references to support your approach.

*R. I added a reference in this regard.*

- Page 14: " In the equation below". Shouldn't be in the equation above?

*R. Yes, thank you! I made the correction.*

- Page 16. Provide some context/details about the use of Age square in your analysis.

*R. I added an explanation in this regard at p.19 (at the point where explaining the effect of age).*

- Typo on page 17: "patters"

*R. I made the correction.*

- Typo on page 19: "detoted"

*R. I made the correction.*

- Page 19: Please, provide clarification, details for CTT

*R. The ICC is described at p.15 (just above Fig.2).*

- Page 21: is TV missing from Fig 2? If so, there is inconsistency with comments and table 2 on Page 20.

*R. I made the correction.*

- Page 25: By reading the following "However, the NATO membership is not a seen as an appropriate foreign policy measure for Moldova, not by Moldovans, and nor by Gagauz people, and one explanation could be the strong Russian opposition (Schelegel, 2018).
Aren't Gagauz people Moldovan citizens? The paragraph could imply a different interpretation. Please, clarify.

*R. I introduced a new paragraph providing more information about the Gaguz people, just above the paragraph cited by reviewer.*

- Typo on page 25: "are in opposition with the all policy" Please, remove 'the'

*R. I made the correction.*

- Page 26: The information provided about attitudes toward refugees is almost negligible. Add more text regarding refugees in Moldova.

*R. I added more text regarding refugees in Moldova.*
